# Results from the second WHO external quality assessment for the molecular detection of respiratory syncytial virus, 2019–2020

**DOI:** 10.1111/irv.13073

**Published:** 2023-01-18

**Authors:** Thomas Williams, Sandra Jackson, Ian Barr, Shabana Bi, Jinal Bhiman, Joanna Ellis, Anne von Gottberg, Stephen Lindstrom, Teresa Peret, Sanjiv Rughooputh, Mariana Viegas, Siddhivinayak Hirve, Maria Zambon, Wenqing Zhang, WHO RSV Surveillance Group, Ndongo Dia, Norosoa Razanazatovo, Ajaeb Dakhilalla M. H. Al‐Nabet, Abdinasir Abubakar, Almiro Tivane, Amal Barakat, Amel Naguib, Ammar Aziz, Andrea Vicari, Ann Moen, Arunkumar Govindakarnavar, Aron Hall, Badarch Darmaa, Bastien Nathalie, Belinda Herring, Braulia C. Caetano, Brett Whittaker, Elsa Baumeister, Emmanuel Nakouné, Erica Guthrie, Francis Inbanathan, Harish Nair, Harry Campbell, Herve A. Kadjo, Hicham Oumzil, Jean‐Michel Heraud, Joshua A. Mott, Joyce Namulondo, Juliana Leite, Karen Nahapetyan, Lubna Al Ariqi, Mahmoud Hamad Ibraheem Gazo, Mandeep Chadha, Maria Pisareva, Marietjie Venter, Marilda M. Siqueira, Mayan Lumandas, Mbayame Niang, Mona Albuaini, Muhammad Salman, Steve Oberste, Padmini Srikantiah, Patrick Tang, Paula Couto, Peter Smith, Peter Valentine Coyle, Philippe Dussart, Phuong Nam Nguyen, Pilailuk Akkapaiboon Okada, Pushpa Ranjan Wijesinghe, Reuben Samuel, Richard Brown, Richard Pebody, Rodrigo Fasce, Runa Jha, Stephen Lindstrom, Sue Gerber, Varsha Potdar, Xiaomin Dong, Yi Mo Deng

**Affiliations:** ^1^ University of Edinburgh Edinburgh UK; ^2^ World Health Organization Geneva Switzerland; ^3^ Peter Doherty Institute for Infection and Immunity WHO Collaborating Centre for Reference and Research on Influenza, Victorian Infectious Disease Reference Laboratory (VIDRL) Melbourne Victoria Australia; ^4^ Department of Microbiology and Immunology University of Melbourne Melbourne Victoria Australia; ^5^ United Kingdom Health Security Agency (UKHSA) London UK; ^6^ United Kingdom National External Quality Assessment Service (UK NEQAS) for Microbiology London UK; ^7^ Centre for Respiratory Diseases and Meningitis National Institute for Communicable Diseases (NICD) of the National Health Laboratory Service Johannesburg South Africa; ^8^ School of Pathology, Faculty of Health Sciences University of the Witwatersrand Johannesburg South Africa; ^9^ Respiratory Virus Branch, Division of Viral Diseases Centers for Disease Control and Prevention Atlanta Georgia USA; ^10^ Division of Infectious Diseases, Department of Internal Medicine University of Texas Medical Branch Galveston Texas USA; ^11^ Institute for Human Infections and Immunity University of Texas Medical Branch Galveston Texas USA; ^12^ Virology Laboratory Ricardo Gutiérrez Children's Hospital Buenos Aires Argentina; ^13^ National Council for Scientific and Technological Research (CONICET) Buenos Aires Argentina; ^14^ Qatar‐HAMAD‐NIC, Department of Laboratory Medicine and Pathology Hamad Medical Corporation Doha Qatar; ^15^ Central Public Health Laboratory Ministry of Health Cairo Egypt; ^16^ Viral Isolation Laboratory, Department of Technological Platforms National Institute of Health/MISAU Marracuene Mozambique; ^17^ Eastern Mediterranean Regional Office World Health Organization Cairo Egypt; ^18^ Pan American Health Organization Washington District of Columbia USA; ^19^ WHO Country Office Kathmandu Nepal; ^20^ Division of Viral Diseases, Respiratory Viruses Branch Centers for Disease Control and Prevention (CDC) Atlanta Georgia USA; ^21^ Virology Laboratory National Center for Communicable Diseases Ulaanbaatar Mongolia; ^22^ Influenza and Respiratory Viruses Section, National Microbiology Laboratory Public Health Agency of Canada Winnipeg Manitoba Canada; ^23^ African Region Office World Health Organization Brazzaville Congo; ^24^ Oswaldo Cruz Foundation Respiratory Virus Laboratory FIOCRUZ Rio de Janeiro Brazil; ^25^ Respiratory Diseases Branch (RDB), Division of Bacterial Diseases (DBD) Centers for Disease Control and Prevention (CDC) Atlanta Georgia USA; ^26^ Respiratory Virosis Service, Virology Department INEI‐ANLIS Dr. Carlos G Malbrán Buenos Aires Argentina; ^27^ Epidemiological Surveillance Pasteur Institute of Bangui Bangui Central African Republic; ^28^ International Reagent Resources (IRR), Centers for Disease Control and Prevention (CDC) Atlanta Georgia USA; ^29^ South‐East Asia Region Office World Health Organization New Delhi India; ^30^ Medical School The University of Edinburgh Edinburgh UK; ^31^ Usher Institute of Population Health Research and Informatics University of Edinburgh Edinburgh UK; ^32^ Department of Epidemic Viruses Pasteur Institute of Ivory Coast Abidjan Côte d'Ivoire; ^33^ Virology Department, Manager of National Influenza Center National Institute of Hygiene, Ministry of Health Rabat Morocco; ^34^ National Influenza Laboratory Institut Pasteur de Madagascar Antananarivo Madagascar; ^35^ US CDC Thailand sub‐regional office Nonthaburi Thailand; ^36^ Uganda Virus Research Institute Entebbe Uganda; ^37^ Western Pacific Region Office World Health Organization Manila Philippines; ^38^ Eastern Mediterranean Region Office World Health Organization Cairo Egypt; ^39^ National Institute of Virology Indian Council of Medical Research Pune India; ^40^ Laboratory of Molecular Virology Smorodintsev Research Institute of Influenza St. Petersburg Russian Federation; ^41^ Center for Viral Zoonosis, Department of Medical Virology University of Pretoria Pretoria South Africa; ^42^ Laboratorio de Virus Respiratorios, IOC/FIOCRUZ Rio de Janeiro Brazil; ^43^ Department of Health Research Institute for Tropical Medicine Muntinlupa Philippines; ^44^ Virology Department National Influenza Centre, Pasteur Institute of Dakar Dakar Senegal; ^45^ National Influenza Centre, Research Laboratory Rafic Hariri University Hospital Beirut Lebanon; ^46^ Central of Public Health Laboratory National Institute of Health Islamabad Pakistan; ^47^ Bill and Melinda Gates Foundation Seattle Washington USA; ^48^ Sidra Medicine Doha Qatar; ^49^ MRC International Statistics and Epidemiology Group London School of Hygiene and Tropical Medicine London UK; ^50^ Department of Medical Sciences Respiratory virus section, Thai NIC, National Institute of Health Nonthaburi Thailand; ^51^ Regional Office for Europe World Health Organization Copenhagen Denmark; ^52^ Sub‐department of Viral Diseases Institute of Public Health of Chile Santiago Chile; ^53^ National Public Health Laboratory, Department of Health Services Ministry of Health and Population Kathmandu Nepal

**Keywords:** laboratory diagnostics and systems, respiratory diseases, strengthening laboratory capacity for infectious disease control

## Abstract

**Background:**

External quality assessments (EQAs) for the molecular detection of human respiratory syncytial virus (RSV) are necessary to ensure the standardisation of reliable results. The Phase II, 2019–2020 World Health Organization (WHO) RSV EQA included 28 laboratories in 26 countries. The EQA panel evaluated performance in the molecular detection and subtyping of RSV‐A and RSV‐B. This manuscript describes the preparation, distribution, and analysis of the 2019–2020 WHO RSV EQA.

**Methods:**

Panel isolates underwent whole genome sequencing and in silico primer matching. The final panel included nine contemporary, one historical virus and two negative controls. The EQA panel was manufactured and distributed by the UK National External Quality Assessment Service (UK NEQAS). National laboratories used WHO reference assays developed by the United States Centers for Disease Control and Prevention, an RSV subtyping assay developed by the Victorian Infectious Diseases Reference Laboratory (Australia), or other in‐house or commercial assays already in use at their laboratories.

**Results:**

An in silico analysis of isolates showed a good match to assay primer/probes. The panel was distributed to 28 laboratories. Isolates were correctly identified in 98% of samples for detection and 99.6% for subtyping.

**Conclusions:**

The WHO RSV EQA 2019–2020 showed that laboratories performed at high standards. Updating the composition of RSV molecular EQAs with contemporary strains to ensure representation of circulating strains, and ensuring primer matching with EQA panel viruses, is advantageous in assessing diagnostic competencies of laboratories. Ongoing EQAs are recommended because of continued evolution of mismatches between current circulating strains and existing primer sets.

## 1 INTRODUCTION

Human respiratory syncytial virus (HRSV, henceforth referred to as RSV) is an RNA virus that in 2019 was estimated to cause 33.0 million serious respiratory infections in children under 5 years of age annually, resulting in 3.6 million hospitalisations and 101 400 deaths^1^ globally. There is a paucity of data regarding the true burden of RSV in low‐ and middle‐income countries (LMICs). A Global RSV Surveillance Program was therefore launched by the World Health Organization (WHO) Global Influenza Program in 2016 initially in 14 countries across the six WHO regions to establish a global surveillance infrastructure to monitor the incidence and prevalence of RSV, particularly in LMICs, and to provide standardised scientific data to guide the introduction of RSV vaccines or other immunoprophylaxis/treatments once they become available.^2^ Participating laboratories were selected from those within the Global Influenza Surveillance and Response System (GISRS)^3^ with a history of successful performance in the molecular detection of influenza viruses. One of the aims of the initial phase of the global surveillance was to evaluate and standardise WHO recommended and other existing RSV molecular tests to enable the accurate detection of RSV by laboratories in all six WHO regions. To achieve this, in 2016, the first External Quality Assessment exercise for the molecular detection of RSV was successfully developed, launched, and completed by the 14 participating national laboratories.^4^ Following the successful implementation of Phase I in 2018, this was expanded to 26 countries in Phase II, each with a national laboratory participating in the Global RSV surveillance Program (Table S1). This study describes the second of the WHO External Quality Assessments (EQA) performed during 2019–2020 for the molecular detection of RSV.

## 2 METHODS

The objective of the RSV EQA 2019–2020 was to assess laboratory proficiency in the molecular detection and subtyping of RSV into the two main subtypes, RSV‐A and RSV‐B. In the preparation of an EQA, it is important that the composition of panels is representative not only of historical but also of current circulating RSV strains, and that primers and probes of assays are sufficiently sensitive to the target sites to be able to detect currently circulating lineages. In addition, the panel should provide a resource of reference virus isolates with known sequences for laboratories globally. In the development of an improved EQA for the molecular detection of RSV, in 2019 the Global Influenza Program collaborated with four WHO RSV Reference Laboratories: the Victorian Infectious Diseases Reference Laboratory, Victorian Infectious Disease Reference Laboratory (VIDRL), Melbourne, Australia; the United Kingdom Health Security Agency (UKHSA), previously Public Health England (PHE); the National Institute for Communicable Diseases, Johannesburg, South Africa (NICD); and the Centers for Disease Control and Prevention, Atlanta, USA (CDC). Following the selection of candidate viruses, the EQA panel was manufactured by the UK National External Quality Assessment Service (UK NEQAS) and distributed to participating countries.

**FIGURE 1:**
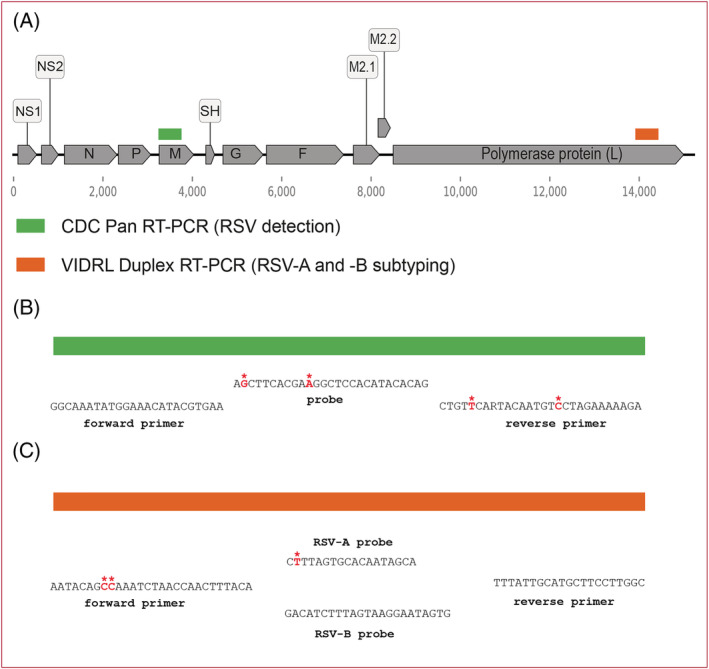
(A) Respiratory syncytial virus (RSV) genome schematic organisation, and location of the CDC detection (M gene) and Victorian Infectious Disease Reference Laboratory (VIDRL) (L gene) subtyping primers/probes. (B) Mismatches between the CDC detection pan RT‐PCR primers/probe and the M gene for the 2019 EQA sequences. The forward primer matches all 8 RSV‐A and RSV‐B M gene sequences. The probe matches all the RSV‐B samples but has two mismatches shared by all the RSV‐A sequences. The reverse primer matches with all the RSV‐A sequences, but there are 2 mismatches with the RSV‐B sequences. (C) Mismatches between the VIDRL duplex subtyping primers/probe and the L gene for the 2019 EQA sequences. The forward primer shows two mismatches with all the available RSV‐B samples, at nucleotide positions 8 and 9. The RSV‐B detection probe and reverse primer matches all four sequences exactly. The forward primer matches with all three RSV‐A available sequences, as does the reverse primer. The RSV‐A detection probe shows one mismatch (shared by all the available RSV‐A sequences). Mismatched nucleotides shown in bold red with overlying asterisk (*); primer sequences aligned with gene sequence, using reverse complement if required.

The selection of the EQA panel viruses was made by the four WHO RSV Reference Laboratories and consisted of 12 lyophilised samples. These consisted of 10 RSV positive samples, with one historical and nine recent RSV circulating viruses collected from 2015 to 2019 (including both RSV‐A and RSV‐B strains), and two RSV negative samples. One of the negative samples contained freeze‐dried sample matrix only, whereas the second contained an influenza B isolate. The historical isolate chosen was the A2 strain^5^ deposited in 2007 in the UKHSA National Collection of Pathogenic Viruses.^6^ Two freeze‐dried controls, one RSV‐A and one RSV‐B isolate, for use by national laboratories participating in the EQA were also included (see Section 3 for details of all isolates included in the panel). These labelled samples were included to allow participating laboratories to develop, evaluate or improve the performance of assays for the detection, and typing of RSV. The EQA panel was also tested by the four WHO RSV Reference Laboratories and evaluated against primer/probe sets developed by the CDC^7^ that target the RSV M gene, and VIDRL^8^ that targets the RSV L gene (Figure 1A).

### 2.1 Globally representative whole genome sequencing datasets for RSV‐A and RSV‐B

Reference datasets of RSV WGS for both subtypes were created. For RSV‐A, a total of 1010 complete genomes and for RSV‐B a total of 633 complete genomes, available up to 3 May 2019, were downloaded from GenBank, aligned with MUSCLE,^9^ curated and used for the matching analyses of the CDC and VIDRL primers and probes. The criteria used to curate the sequences include removing sequences with any ambiguous nucleotides (“N” s), and/or sequences with deletions causing frameshifts. Sequences with incorrect RSV subtype annotation were identified and added to the database of correct subtype.

### 2.2 Sharing and selection of viruses for EQA panel

Each of three WHO RSV Reference Laboratories (NICD, PHE and VIDRL) submitted RSV‐A and RSV‐B isolates for consideration. Viruses shared for consideration were collected by the reference laboratories during the years 2015, 2017, 2018 and 2019. To include a relatively representative set of circulating sequences, the lineage for each strain was assigned using two approaches; the first was based on the new genotyping classification developed by Goya et al.^10^ and a standard consensus approach. G‐gene ectodomains from the reference sequences were aligned with MUSCLE.^9^ jModelTest (2.1.6)^11^ was used to determine the most suitable evolutionary model for the set of the analysed sequences. Phylogenetic trees were inferred using IQ‐TREE (1.6.7)^12^ for maximum likelihood (ML) analysis and MrBayes (3.2.1)^13^ for Bayesian analysis, with the appropriate statistical branch support (ultrafast bootstrap or posterior probabilities, respectively). Consensus trees were visualised with Figtree (1.4.0).^14^ P‐distances were calculated with MEGA 7^15^ for the G‐gene ectodomain to characterise the most genetically distinct isolates for inclusion in the panel.

An in silico analysis was conducted to ensure that the sequences of all RSV isolates shared matched the recommended primer/probe sets. Isolate sequences were aligned using MAFFT (7.305)^16^ and then manually edited in AliView (1.28)^17^ to retain the primer binding sequences and isolates tested against the recommended WHO CDC RSV detection and VIDRL subtyping assays. Sequences were named in a standardised manner using the approach recently proposed by Salimi et al.^18^

### 2.3 Preparation of panel by NEQAS

Viruses included in the EQA panel were propagated in Hep‐2 cells (a Hela derivative, ECACC 86030501) in growth media (Eagle Minimum Essential Medium‐Ref Sigma M4655‐containing Earle's salt, L‐glutamine and sodium bicarbonate supplemented with 10% fetal bovine serum, Lonza Bioscience ref DE 14‐801F), harvested, and then used to prepare the EQA specimens that were subsequently lyophilised. EQA specimens were designed to cover a wide range of concentrations. Expected Ct values were calculated on the dilutions of original virus stock using a UK NEQAS in‐house protocol. Five vials from each specimen were randomly selected for quality checks for sterility, stability, and other predistribution testing requirements. They were also tested in‐house by a commercial semiquantitative RT‐PCR assay for the relevant targets to verify the calculated Ct value and sent to an external UK reference laboratory for confirmation of content. On successful completion of quality control checks, the specimens were labelled and packed for dispatch the first week of December 2019. The panels were stored and shipped at room temperature.

### 2.4 Development of qualitative survey

The purpose of the survey was to assess the quality of the WHO RSV surveillance system to ensure the data collected by participating laboratories from all countries met set targets and standards. As such the survey was designed by UK NEQAS and the Reference Laboratory Working Group to collect information on general laboratory practices, amplification platforms, nucleic acid extraction methods, RSV molecular detection and subtyping assays used in the EQA. Survey reporting requirements were used to custom design the UK NEQAS website for reporting of information.

### 2.5 Reconstitution of panel and reporting of results

Participants were required to reconstitute the EQA specimens in 1.2 ml of sterile molecular grade water. For nucleic acid extraction protocol and molecular testing, participants were instructed to follow their existing laboratory protocols/kits instructions. Laboratories were advised to store the lyophilised specimens preferably at 4°C. Once reconstituted, samples were to be extracted, and RNA was stored at −80°C for long‐term use.

Countries were given the option to use the RSV singleplex or multiplex RT‐qPCR assays provided by the CDC, USA,^7^ or VIDRL Australia,^8^ respectively, for commercial assays or in‐house assays. Results for the EQA from all assays were required to be reported through the UK NEQAS online platform. Distribution of the panel by UK NEQAS was made to 28 laboratories in 26 countries and commenced in December 2019 with the expected closing date and return of results to be by the end of February 2020. However, it was subsequently agreed that an extension of the closing date to September 2020 be approved given the staffing issues, border closures and shipping challenges of the COVID‐19 pandemic.

### 2.6 Scoring of results

A scoring system was established after consultation between UK NEQAS and the WHO Reference Laboratories (Table 1). For the 12 EQA specimens, participants could score a maximum cumulative score of 24. Interpretation of the scores was as follows: all correct (24/24) was interpreted as a good performance, scores between 22–23 as acceptable; 20–21 as satisfactory; 17–19 as poor; and 16 or less as unacceptable.

**TABLE 1:** Scoring system for the 2019–2020 WHO RSV EQA

Description	Report text	Score
RSV identified	RSV	1
RSV‐A/B identified	RSV‐A/B	1
RSV negative	RSV negative	2
No virus	No virus	0
RSV wrong subtype	RSV wrong subtype	0
Indeterminate	Indeterminate	0
Named virus other than that specified	Named virus	0
RSV + an additional pathogen	Additional pathogen	0

Laboratory results were first analysed by UK NEQAS using expected Ct values for detection and subtyping as determined by UK NEQAS and an external UK Reference Laboratory. The expected and scoring of submitted results were reported to participating laboratories through the NEQAS platform. Additional analysis was performed using results from UK NEQAS and the four RSV Reference Laboratories to determine the expected Ct as acceptable sensitivity with comparison of the coefficient of variation (CV) of participating laboratories with those of Reference Laboratories/UK NEQAS. Inter‐laboratory performance variability was evaluated using expected Ct values and ranges within two standard deviations (*SD*) and the CV.

## 3 RESULTS

### 3.1 Selection of isolates for the EQA

Eleven isolates were submitted for consideration of inclusion in the EQA: 4 (2 RSV‐A, 2 RSV‐B) by PHE (now UKHSA), 4 (2 RSV‐A, 2 RSV‐B) by VIDRL and 3 by NICD (1 RSV‐A, 2 RSV‐B) (Table S2). Nine RSV strains, the most diverse based on G gene P‐distances, were included in the final panel (Table 2) in addition to the historical control.

**TABLE 2:** RSV isolate composition of the 2019–2020 WHO RSV EQA panel

Sample number	Sample designation of RSV isolates	Subtype	GISAID accession ID
1	HRSV/A/England/174460397/2017	A	EPI_ISL_732338
2	HRSV/A/Australia/VIC‐RCH010/2018	A	EPI_ISL_1834085
3	HRSV/A/Australia/VIC‐VIDRL002/2018	A (control)	EPI_ISL_4602779
4	HRSV/A/South Africa/NICD‐R06229/2019	A	EPI_ISL_9003918
5	HRSV/B/England/154680653/2015	B	EPI_ISL_4848359
6	HRSV/B/England/180440410/2018	B	EPI_ISL_732354
7	HRSV/B/Australia/VIC‐VIDRL003/2015	B (control)	EPI_ISL_4569432
8	HRSV/B/South Africa/NICD‐R05898/2019	B	EPI_ISL_9003919
9	HRSV/A/England/0709161v/2007	A	EPI_ISL_11901829
10	HRSV/B/South Africa/NICD‐R06224/2019	B	EPI_ISL_9003920
11	RSV negative specimen (freeze‐dried matrix only)	NA	NA
12	RSV negative specimen (Influenza B B/Phuket/3073/2013)	NA	**EPI_ISL_168822**

*Note*: Two labelled controls, one RSV‐A and one RSV‐B (sample Numbers 3 and 7, respectively) were also provided to participating laboratories as part of the panel in order to facilitate implementation of RSV detection and typing assays.

### 3.2 Genotyping of EQA isolates

Using the standard approach to genotyping, the four non‐historical RSV‐A isolates selected for the RSV EQA 2019 were ON1‐like strains, and the 5 RSV‐B isolates were BA‐like ones, with the previously described 72‐nt and the 60‐nt duplications, respectively, distinguishing them from previously circulating lineages. Within the new genotyping system proposed by Goya et al.,^10^ the RSV‐A strains clustered with the GA2.3.5 lineage within the GA2 genotype; and all EQA RSV‐B strains clustered with the GB5.0.5a lineage within the GB5 genotype.

### 3.3 CDC detection and VIDRL subtyping primers/probes: Matching to EQA strains

Whole genome sequences are available for all isolates included in the RSV EQA 2019 except the historical A2 strain, and their M gene sequences were matched to the CDC detection primers/probes. A maximum of two mismatches was found per primer/probe (Figure 1B). The VIDRL subtyping primers/probe were matched to the available L gene sequences (Figure 1C). Of note, one RSV‐A (HRSV/A/South Africa/NICD‐R06229/2019) and one RSV‐B (HRSV/B/South Africa/NICD‐05898/2019) had incomplete coverage of the L gene. Again, a maximum of two mismatches were found per primer/probe. The RSV‐A detection probe (RSV‐L1‐A‐P) did not match any of the RSV‐B sequences; conversely the RSV‐B detection probe (RSV‐L1‐B‐P) did not match any of the RSV‐A sequences, ensuring there was no possibility of cross reactions. In summary, the sequences of the EQA strains showed a high degree of matching to the two primers/probe sets recommended by the WHO for RSV detection and subtyping.

### 3.4 Matching of detection and subtyping primers to globally circulating RSV strains

The CDC detection and VIDRL subtyping primers were matched to the curated database of 1010 RSV‐A and 633 RSV‐B WGS (Table 3). The sequences with the highest number of mismatches over the CDC detection primers/probe was found to be a strain from Jordan (HRSV/A/Jordan/JOR‐D3721/2013, GISAID ID EPI_ISL_2588376, 2013) with four mismatches across the probe and reverse primer. For the VIDRL RSV‐A subtyping primers/probes, 18 sequences with a total of three mismatches across the primers/probe were found. For RSV‐B, the most divergent sequence was HRSV/B/Nicaragua/NIC‐7309‐04/2013 (GISAID ID EPI_ISL_2577425, 2013) which had five mismatches across the forward primer and probe. For this strain, none of the mismatches were found to be in the final five nucleotides of the 3′ of the VIDRL RSV‐B primer/probe sequence and can therefore be inferred to be unlikely to significantly impact on primer probe binding. In summary, although mismatches were found in both RSV‐A and RSV‐B strains circulating globally, it was predicted that all globally circulating strains would be correctly identified by the detection and subtyping assays recommended by the WHO.

**TABLE 3:** Primers and probe mismatches to current circulating RSV‐A and RSV‐B strains

Primer set	Primer/probe	Primer/probe sequence (5′– > 3′)	Mismatching primer/probe positions (5′– > 3′) in one or more circulating strains
CDC Pan RSV Assay	forward primer	GGCAAATATGGAAACATACGTGAA	16 (RSV‐A)
CDC Pan RSV Assay	probe	CTGTGTATGTGGAGCCTTCGTGAAGCT	2,8,11,14,17,26 (RSV‐A)
CDC Pan RSV Assay	reverse primer	TCTTTTTCTAGGACATTGTAYTGAACAG	3,6,9,11,12 (RSV‐A) 3,5,6,11,12,24 (RSV‐B)
VIDRL Duplex subtyping assay	forward primer	AATACAGCCAAATCTAACCAACTTTACA	6,7,9,12,15,18,24 (RSV‐A) 3,8,9,11,15,17,21,27 (RSV‐B)
VIDRL Duplex subtyping assay	RSV‐A probe	6FAM‐TGCTATTGTGCACTAAAG‐MGBNFQ	2,11,13,14,16,17 (RSV‐A)
VIDRL Duplex subtyping assay	RSV‐B probe	VIC‐CACTATTCCTTACTAAAGATGTC‐MGBNFQ	2,5,14,16,17 (RSV‐B)
VIDRL Duplex subtyping assay	reverse primer	GCCAAGGAAGCATGCAATAAA	10,13,17,20 (RSV‐A) 8,13,17 (RSV‐B)

### 3.5 RSV detection and subtyping results

The survey was opened 2 December 2019; first results were submitted on 16 January 2020, and results were collated on 16 November 2020. Twenty‐eight laboratories submitted results for the detection of RSV, and 25 for the subtyping results.

For the 12‐specimen panel, the contents (RSV positive or negative) were correctly identified in 332/336 cases (98.8%). All participating laboratories identified the 10 RSV positive specimens (280/280). Of 28 participants who reported on the negative specimen containing only freeze‐dried matrix, 2/28 (7.1%) incorrectly reported the presence of RSV and obtained a score of 0 on this sample. Twenty‐seven out of 28 laboratories reported results for the second RSV negative sample containing an influenza B virus. Three out of 27 participants (11.1%) correctly identified this specimen as influenza B; and 22 out of 27 (81.4%) reported this as negative for RSV and did not test the sample for influenza. Two participants (7.4%) reported this specimen as being positive for RSV and therefore obtained a score of zero.

For subtyping, 25 out of 28 laboratories reported results. Of these, one laboratory reported an RSV‐B specimen as untypable, meaning samples were correctly typed in 99.6% of instances (249/250). Scores were allocated to the 25 laboratories that had submitted subtyping results (Table 4). Of these, 21 obtained a score of *good*, and 4 obtained a score of *acceptable*.

**TABLE 4:** Scores achieved by the 25 laboratories conducting RSV subtyping in the 2019–2020 WHO RSV EQA

Score	Interpretation	Number of laboratories
24	*Good*	21
22–23	*Acceptable*	4^a^
20–21	*Satisfactory*	0
17–19	*Poor*	0
<17	*Unacceptable*	0
TOTAL		25

^a^ One of these four laboratories incorrectly typed a specimen and therefore lost 1 point; the remaining three incorrectly identified an RSV negative sample as RSV positive and lost 2 points.

### 3.6 Sensitivity of RSV detection: Ct values

To analyse diagnostic sensitivity, Ct values obtained from the four RSV Reference Laboratories and UK NEQAS and RSV Ct values from participating laboratories were calculated. Further analyses compared the RSV Ct values from the participating laboratories with the mean Ct value of the Reference Laboratories (Table S3). Twenty‐seven laboratories submitted Ct results: the 4 WHO RSV Reference Laboratories and 23 participating National Laboratories. Among the 23 participating national laboratories that submitted Ct results, 87% (200/230) of the mean Cts reported per sample were within 2 *SD* of the mean expected Ct values for detection calculated by Reference Laboratories (Table S3). However, 13% (30/230) of Ct values reported showed variation >2 *SD* above the Reference Laboratory/UK NEQAS mean Ct value for detection. By investigating these results, there were two participating laboratories that consistently reported Cts of 2 *SD* or above the other laboratories for 9 of the 10 samples. Both these laboratories had been previously enrolled in the pilot and utilised the widely used QIAmp viral RNA extraction method. One laboratory used the ABI 7500 Amplification Platform, and another used the CFX96 Touch Amplification Platform, both of which were used by other laboratories participating in the EQA. One of the laboratories used the CDC detection RT‐PCR assay, and another used an in‐house assay.

Participant results showed CVs between 0.10 and 0.13 compared with the CV range of 0.04 to 0.08 for Reference Laboratories (Table S3). This suggests that the participating laboratories had lower sensitivities for the detection of RSV than the Reference Laboratories and UK NEQAS.

Results from the qualitative survey consisted the following: extraction, amplification platforms and detection and typing assays used by National Laboratories.

The QIAamp Viral RNA kit was the most frequently used nucleic acid extraction method (Table S4). Eight amplification platforms were used by participants in the 2019 EQA: the ABI 7500 was the platform most frequently used followed by the BioRad, Rotor‐Gene and Cepheid GeneXpert. A total of 10 detection methods were used, with the CDC pan RSV detection assay^7^ and multiplex assay^19^ being the most frequently used methods, followed by the Cepheid Gene Xpert and the VIDRL duplex assay.^8^ The most common typing assay used was the CDC multiplex RSV Assay.^19^

## 4 DISCUSSION

The WHO EQA 2019–2020 for the detection of RSV by real‐time RT‐PCR was completed successfully by the 28 laboratories participating in Phase II of WHO RSV Surveillance Program, and for subtyping by 25 laboratories. The extension of the duration of the EQA was permitted due to shipping challenges and surge capacity burden associated with the COVID‐19 pandemic as the laboratories participating in Phase II were also the responsible laboratories for the detection of SARS‐CoV‐2. Despite the positive outcome obtained by the participant laboratories (98.8% correct identification within the expected RSV Ct range), two laboratories consistently reported results with RSV Ct values 2 *SD* above the mean value of other laboratories. As the extraction methods, platforms, amplification kits and detection assays used were not unique to these sites, extraction methods (automated/manual) and laboratory techniques and analysis may have contributed to a decrease in sensitivity.

The CDC detection assay provided for this EQA^7^ targets the M gene and the VIDRL assay the L gene.^8^ WGS of the isolates used in the RSV EQA 2019–2020 showed no mutations likely to impact on the effectiveness of these primer/probe sets used as part of Phase II. In addition, both the CDC and VIDRL primer/probe sets were matched against the sequences available on GenBank. The highest number of mismatches across probe and primer for the CDC assay was 4, and for the VIDRL typing assay there were samples with 3 and 5 mismatches across the primers/probe for RSV‐A and B respectively. Despite the identification of some mismatches, they were judged insufficient to affect the assays' ability to detect the RSV strains in the panel. However, the described mismatches might have slightly impacted assay sensitivity. Of note, a third assay (a CDC multiplex assay targeting the N gene^19^) was provided to participating laboratories from February 2020; a formal analysis of primer/probe matching was not conducted as part of the design of this EQA, and is therefore a limitation of this study.

Genomic variability in RSV is especially relevant in laboratories that have been using in‐house developed assays. A recent survey^20^ found that the N gene is the most common RT‐PCR target for RSV detection assays, and therefore, ongoing review of WGS of circulating RSV strains is required, as there is the potential for resultant genetic drift which may impact the effectiveness of such diagnostics, the accuracy of which is needed for the RSV detection, management and outbreak investigation. The importance of ongoing surveillance of the entire RSV genome is highlighted by a recent example where mutations in the N gene of circulating RSV‐B strains affected the efficacy of a custom rRT‐PCR assay for the detection of RSV.^21^

The objective of the first WHO EQA^4^ was to ensure that laboratories and countries enrolled into the RSV pilot project could acquire suitable surveillance samples and implement a CDC real‐time RT‐PCR assay for RSV detection. The objectives of the second EQA were to ensure that an expanded network of countries/laboratories using a greater diversity of laboratory methods could detect recently circulating strains, known to be genetically diverse from the selected reference strains. The performance of laboratories at RSV detection showed an improvement in the second EQA compared with the first. There were fewer errors and closer concordance between observed and expected results. This may reflect greater familiarity with the protocols as well as more experience gained by laboratory workforce during the expansion of global molecular testing during the COVID‐19 pandemic.

Overall, the pandemic has had a significant impact on the Phase II of the WHO RSV Surveillance Program. In many countries during the Covid‐19 pandemic, RSV circulation largely disappeared or was greatly reduced, meaning few RSV positive cases were present with a consequent reduction in sampling,^22^ while in others, the urgent need for surveillance of SARS CoV‐2 has diverted resources away from RSV surveillance. Previous patterns of circulation of RSV may continue to be seriously disrupted for some years to come,^23^ although co‐infection with SARS‐CoV‐2 and RSV has been documented^24^, ^25^ and may increase with time. The fact that both SARS‐CoV‐2 and RSV present initially with similar clinical manifestations highlights the importance of accurately identifying RSV with implications for cost‐effective management and control of both diseases.^26^

## 5 CONCLUSIONS

The RSV EQA 2019–2020 for countries participating in the Phase II of the WHO Global RSV Surveillance Program showed that the laboratories performed at high standards. Some laboratories reported RT‐PCR Ct values more than 2 *SD* above the mean with higher CVs, compared with expected reference ranges, indicating possible decreased sensitivity of some methods. Updating the composition of RSV molecular EQAs with current circulating strains is important for the accurate testing of clinical samples. This work is fundamental to the WHO RSV Surveillance Program, which aims to better understand the impact and burden of RSV globally and which will enable support for the development of vaccines and therapeutics. The principles used for the preparation of the WHO RSV EQA are applicable to the development of other molecular assays. A third WHO RSV EQA is being conducted in 2021–2022.

## CONFLICT OF INTEREST

No conflict of interest is declared by authors.

## AUTHOR CONTRIBUTIONS

Sandra Jackson, Thomas Williams and Wenqing Zhang gave substantial contributions to the conception or design of the work, the acquisition, analysis, or interpretation of data for the work, the drafting the work and revising it critically for important intellectual content and final approval of the version to be published.

The authors agree to be accountable for all aspects of the work in ensuring that questions related to the accuracy or integrity of any part of the work are appropriately investigated and resolved.

Ian Barr, Shabana Bi, Jinal Bhiman, Joanna Ellis, Anne von Gottberg, Stephen Lindstrom, Teresa Peret, Sanjiv Rughooputh, Mariana Viegas, Siddhivinayak Hirve^2^ and Maria Zambon gave substantial contributions to the conception or design of the work, the acquisition, analysis, or interpretation of data for the work, the drafting the work and revising it critically for important intellectual content and final approval of the version to be published.

The WHO RSV Surveillance Group: This group gave substantial contributions to the acquisition, analysis and interpretation of data for the work; revising the work for important intellectual content and final approval of the version to be published.

### PEER REVIEW

The peer review history for this article is available at https://publons.com/publon/10.1111/irv.13073.

## Supporting information


**Table S1:** Countries and laboratories participating in the 2019–2020 RSV EQA.
**Table S2.** Isolates submitted by Reference Laboratories for inclusion in the 2019–2020 RSV EQA.
**Table S3:** Summary of WHO RSV EQA 2019–2020 variation (mean, SD, and CV) between participant and RSV Reference Laboratories.
**Table S4:** Nucleic acid extraction, amplification, detection and typing assays used by laboratories participating in the 2019–2020 EQA

## Data Availability

Data available on request due to privacy/ethical restrictions.

## APPENDIX A

WHO RSV Surveillance Group.

1. Ajaeb Dakhilalla M H Al‐Nabet (Qatar‐HAMAD‐ NIC, Department of Laboratory Medicine and Pathology, Hamad Medical Corporation, Doha, Qatar)
2. Abdinasir Abubakar (Central Public Health Laboratory, Ministry of Health, Cairo, Egypt)
3. Almiro Tivane (Laboratório de Isolamento Viral, Departmento de Plataformas Tecnológicas, Instituto Nacional de Saúde/MISAU, Mozambique)
4. Amal Barakat (Eastern Mediterranean Regional Office, World Health Organization, Cairo, Egypt)
5. Amel Naguib (Central Public Health Laboratory, Ministry of Health, Cairo, Egypt)
6. Ammar Aziz (The Peter Doherty Institute for Infection and Immunity, WHO Collaborating Centre for Reference and Research on Influenza, VIDRL, Australia)
7. Andrea Vicari (Pan American Health Organization, Washington, DC, USA)
8. Ann Moen (World Health Organization, Geneva, Switzerland)
9. Arunkumar Govindakarnavar (WHO Country Office, Nepal)
10. Aron Hall (Division of Viral Diseases, Respiratory Viruses Branch, Centers for Disease Control and Prevention (CDC), Atlanta, GA, USA)
11. Badarch Darmaa, (Virology Laboratory, National Center for Communicable Diseases, Ulanbaatar, Mongolia)
12. Bastien, Nathalie (Influenza and Respiratory Viruses Section, National Microbiology Laboratory, Public Health Agency of Canada, Winnipeg, MB, Canada)
13. Belinda Herring (African Region Office, World Health Organization, Brazzaville, Republic of Congo)
14. Braulia C. Caetano (Fundação Oswaldo Cruz Laboratório de Vírus Respiratorios FIOCRUZ‐ Rio de Janeiro, Brazil)
15. Brett Whittaker (Respiratory Diseases Branch (RDB), Division of Bacterial Diseases (DBD)
16. Centers for Disease Control and Prevention (CDC), Atlanta, GA, USA)
17. Elsa Baumeister (Servicio Virosis Respiratorias, Departamento Virologia, INEI‐ANLIS Dr. Carlos G Malbrán, Buenos Aires, Argentina)
18. Emmanuel Nakouné (Surveillance épidémiologique Institut Pasteur de Bangui, Central African Republic)
19. Erica Guthrie (International Reagent Resources (IRR), Centers for Disease Control and Prevention (CDC), Atlanta, GA, USA)
20. Francis Inbanathan (South‐East Asia Region Office, World Health Organization, New Delhi, India)
21. Harish Nair (The University of Edinburgh, Medical School, Edinburgh, United Kingdom)
22. Harry Campbell (Usher Institute of Population Health Research and Informatics, University of Edinburgh, Edinburgh, United Kingdom)
23. Herve A. Kadjo Department of Epidemic Viruses, Institut Pasteur de Côte d'Ivoire, Abidjan, Côte d'Ivoire
24. Hicham Oumzil (Virology Department, Manager of National Influenza Center, National Institute of Hygiene, Ministry of Health, Rabat, Morocco)
25. Jean‐Michel Heraud (National Influenza Laboratory, Institut Pasteur de Madagascar)
26. Joshua A. Mott (US CDC Thailand sub‐regional office) Thailand
27. Joyce Namulondo (Uganda Virus Research Institute, Entebbe, Uganda)
28. Juliana Leite (Pan American Health Organization, Washington, DC, USA)
29. Karen Nahapetyan (Western Pacific Region Office, World Health Organization, Manila, Philippines)
30. Lubna Al Ariqi (Eastern Mediterranean Region Office, World Health Organization, Cairo, Egypt)
31. Mahmoud Hamad Ibraheem Gazo (Central of Public Health Laboratory, Ministry of Health, Amman, Jordan)
32. Mandeep Chadha (National Institute of Virology, Indian Council of Medical Research, Pune, India)
33. Maria Pisareva (Laboratory of Molecular Virology, Smorodintsev Research Institute of Influenza, St. Petersburg, Russian Federation)
34. Marietjie Venter (Center for Viral Zoonosis, Department of Medical Virology, University of Pretoria, Pretoria, South Africa)
35. Marilda M Siqueira (Laboratorio de Virus Respiratorios, IOC/FIOCRUZ.Rio de Janeiro, Brazil)
36. Mayan Lumandas (Research Institute for Tropical Medicine‐Department of Health, Muntinlupa City, Philippines)
37. Mbayame Niang (Virology Department, National Influenza Centre, Institut Pasteur de dakar, Dakar, senegal)
38. Mona Albuaini (National Influenza Centre, Research Laboratory, Rafic Hariri University Hospital, Beirut, Lebanon)
39. Muhammad Salman (Central of Public Health Laboratory, National Institute of Health, Islamabad, Pakistan)
40. Ndongo Dia (Virology Department, National Influenza Centre, Institut Pasteur de dakar, Dakar, senegal)
41. Norosoa Razanazatovo (National Influenza Laboratory, Institut Pasteur de Madagascar)
42. Oberste, Steve (Division of Viral Diseases, Respiratory Viruses Branch, Centers for Disease Control and Prevention (CDC), Atlanta, GA, USA)
43. Padmini Srikantiah (Bill and Melinda Gates Foundation, Seattle, WA, USA)
44. Patrick Tang (Sidra Medicine, Al Gharrafa Street, Doha, Qatar)
45. Paula Couto (Pan American Health Organization, Washington, DC, USA)
46. Peter Smith (MRC International Statistics and Epidemiology Group, London School of Hygiene and Tropical Medicine, London, United Kingdom)
47. Peter Valentine Coyle (HAMAD‐ NIC, Department of Laboratory Medicine and Pathology, Hamad Medical Corporation, Doha, Qatar)
48. Philippe Dussart (National Influenza Laboratory, Institut Pasteur de Madagascar)
49. Phuong Nam Nguyen (Western Pacific Region Office, World Health Organization, Manila, Philippines)
50. Pilailuk Akkapaiboon Okada (Respiratory virus section, Thai NIC, National Institute of Health, Department of Medical Sciences, Thailand)
51. Pushpa Ranjan Wijesinghe (South‐East Asia Region Office, World Health Organization, New Delhi, India)
52. Reuben Samuel (South‐East Asia Region Office, World Health Organization, New Delhi, India)
53. Richard Brown (South‐East Asia Region Office, World Health Organization, New Delhi, India)
54. Richard Pebody (Regional Office for Europe, World Health Organization, Copenhagen, Denmark)
55. Rodrigo Fasce (Sub‐department of Viral Diseases, Instituto de Salud Pública de Chile, Santiago, Chile)
56. Runa Jha (National Public Health Laboratory, Department of Health Services, Ministry of Health and Population, Teku Kathmandu, Nepal)
57. Stephen Lindstrom (Division of Viral Diseases, Respiratory Viruses Branch, Centers for Disease Control and Prevention (CDC), Atlanta, GA, USA)
58. Sue Gerber (Division of Viral Diseases, Respiratory Viruses Branch, Centers for Disease Control and Prevention (CDC), Atlanta, GA, USA)
59. Varsha Potdar (National Institute of Virology, Indian Council of Medical Research, Pune, India)
60. Xiaomin Dong (The Peter Doherty Institute for Infection and Immunity, WHO Collaborating Centre for Reference and Research on Influenza, VIDRL, Australia)
61. Yi Mo Deng (The Peter Doherty Institute for Infection and Immunity, WHO Collaborating Centre for Reference and Research on Influenza, VIDRL, Australia)
