# Supplementary material for: Results from the second WHO external quality assessment for the molecular detection of respiratory syncytial virus, 2019–2020
Source: Influenza Other Respir Viruses. 2023 Jan 18;17(1):e13073. doi: 10.1111/irv.13073 (PMC9849090; doi:10.1111/irv.13073)
Supplement: Supplementary file 1 — Table S1: Countries and laboratories participating in the 2019–2020 RSV EQA. Table S2. Isolates submitted by Reference Laboratories for inclusion in the 2019–2020 RSV EQA. Table S3: Summary of WHO RSV EQA 2019–2020 variation (mean, SD, and CV) between participant and RSV Reference Laboratories. Table S4: Nucleic acid extraction, amplification, detection and typing assays used by laboratories participating in the 2019–2020 EQA [file IRV-17-e13073-s001.docx]

**Supplementary Table 1: Countries and laboratories participating in the 2019-2020 RSV EQA.**

| **Country** | **Number of laboratories** |
| --- | --- |
| Argentina | 1 |
| Australia | 2 |
| Brazil | 1 |
| Canada | 1 |
| Central African Republic | 1 |
| Chile | 1 |
| Cote d’Ivoire | 1 |
| Egypt | 1 |
| India | 1 |
| Jordan | 1 |
| Lebanon | 1 |
| Madagascar | 1 |
| Mongolia | 1 |
| Morocco | 1 |
| Mozambique | 1 |
| Nepal | 1 |
| Pakistan | 1 |
| Philippines | 1 |
| Qatar | 2 |
| Russian Federation | 1 |
| Senegal | 1 |
| South Africa | 1 |
| Thailand | 1 |
| Uganda | 1 |
| UK | 1 |
| USA | 1 |

**Supplementary Table 2. Isolates submitted by Reference Laboratories for inclusion in the 2019-2020 RSV EQA.**

| **Sample designation of RSV isolates** | **Subtype** | **GISAID Accession ID** | **Included in EQA Panel (Y/N) *** |
| --- | --- | --- | --- |
| HRSV/A/England/174460397/2017 | A | EPI_ISL_732338 | Y |
| HRSV/A/England/174560282/2017 | A | EPI_ISL_10954008 | N |
| HRSV/A/Australia/VIC-RCH010/2018 | A | EPI_ISL_1834085 | Y |
| HRSV/A/Australia/VIC-VIDRL002/2018 | A | EPI_ISL_4602779 | Y |
| HRSV/A/South Africa/NICD-R06229/2019 | A | EPI_ISL_9003918 | Y |
| HRSV/B/England/154680653/2015 | B | EPI_ISL_4848359 | Y |
| HRSV/B/England/180440410/2018 | B | EPI_ISL_732354 | Y |
| HRSV/B/Australia/VIC-VIDRL003/2015 | B | EPI_ISL_4569432 | Y |
| HRSV/B/Australia/VIC-VIDRL003/2018 | B | EPI_ISL_10914987 | N |
| HRSV/B/South Africa/NICD-R05898/2019 | B | EPI_ISL_9003919 | Y |
| HRSV/B/South Africa/NICD-R06224/2019 | B | EPI_ISL_9003920 | Y |

* Y/N = Yes/No

**Supplementary Table 3:** **Summary of WHO RSV EQA 2019-2020 variation (mean, SD, and CV) between participant and RSV Reference Laboratories**.

| RSV subtype | Category | RSV-B | RSV-A | RSV-B | RSV-A | RSV-A |
| --- | --- | --- | --- | --- | --- | --- |
| Sample designation |  | England/154680653/2015 | England/0709161v/2007 | England/180440410/2018 | Australia/VIC-RCH010/2018 | Australia/VIC-VIDRL002/2018 |
| Mean Ct | Participants | 23.32 | 25.50 | 20.43 | 25.26 | 21.70 |
| SD |  | 2.46 | 3.32 | 2.64 | 2.44 | 2.45 |
| CV (SD) |  | 0.11 | 0.13 | 0.13 | 0.10 | 0.11 |
| Mean Ct | Reference Laboratories | 23.41 | 23.47 | 20.41 | 25.66 | 22.29 |
| SD |  | 2.49 | 1.53 | 2.00 | 1.00 | 0.91 |
| Mean Ct + SD |  | 25.90 | 25.00 | 22.41 | 26.65 | 23.20 |
| Mean Ct +2SD |  | 28.39 | 26.53 | 24.42 | 27.65 | 24.12 |
| CV (SD) |  | 0.11 | 0.07 | 0.10 | 0.04 | 0.04 |
| RSV subtype | Category | RSV-A | RSV-A | RSV-B | RSV-B | RSV-B |
| Sample designation |  | South Africa/NICD-R06229/2019 | England/174460397/2017 | Australia/VIC-VIDRL003/2015 | South Africa/NICD-R05898/2019 | South Africa/NICD-R06224/2019 |
| Mean Ct | Participants | 23.24 | 19.90 | 25.85 | 24.51 | 25.59 |
| SD |  | 2.45 | 2.24 | 2.84 | 2.71 | 2.86 |
| CV (SD) |  | 0.11 | 0.11 | 0.11 | 0.11 | 0.11 |
| Mean Ct | Reference Laboratories | 23.10 | 21.13 | 25.56 | 24.25 | 24.80 |
| SD |  | 0.93 | 1.09 | 1.14 | 1.55 | 1.98 |
| Mean Ct + SD |  | 24.04 | 22.23 | 26.69 | 25.80 | 26.78 |
| Mean Ct +2SD |  | 24.97 | 23.32 | 27.83 | 27.35 | 28.75 |
| CV (SD) |  | 0.04 | 0.05 | 0.04 | 0.06 | 0.08 |

**Supplementary Table 4:** **Nucleic acid extraction, amplification, detection and typing assays used by laboratories participating in the 2019-2020 EQA**

| **Nucleic acid extraction assay** | **Name** | **Number** |
| --- | --- | --- |
|  | QiaAmp Viral RNA | 15 |
|  | MagnaPure | 2 |
|  | Cepheid: GeneXpert | 2 |
|  | Qiagen | 1 |
|  | Qiagen: silica column | 1 |
|  | Qiagen: EZ1 | 1 |
|  | PureLink Viral RNA/DNA | 1 |
|  | MagaMAX-96 | 1 |
|  | Other | 4 |
|  |  |  |
| **Amplification platform** | **Name** | **Number** |
|  | ABI: 7500 system | 16 |
|  | Qiagen: Rotor-Gene | 3 |
|  | BioRad:CFX96 Touch | 3 |
|  | Cepheid: GeneXpert | 2 |
|  | AusDiagnostics | 1 |
|  | ABI: 7900 system | 1 |
|  | QuantStudio 7 | 1 |
|  | Other | 1 |
|  |  |  |
| **RSV detection assay** | **Name** | **Number** |
|  | CDC: RSV_RUO-01 multiplex | 12 |
|  | CDC Pan RSV detection assay | 4 |
|  | Real-Time Single target | 3 |
|  | VIDRL: Duplex RSV TaqMan | 2 |
|  | Cepheid: GeneXpert | 2 |
|  | AusDiagnostics | 1 |
|  | PHE: RSV-A/B, hMPVA/B | 1 |
|  | FTD FLU/HRSV | 1 |
|  | In house | 1 |
|  | Other | 1 |
|  |  |  |
| **Subtyping assays** | **Name** | **Number** |
|  | CDC: RSV_RUO-01 multiplex | 12 |
|  | VIDRL: Duplex RSV TaqMan | 2 |
|  | AusDiagnostics | 1 |
|  | PHE: RSV-A/B, hMPVA/B | 1 |
|  | Information not provided | 10 |
